# Management of COVID-19-Associated Acute Respiratory Distress Syndrome (ARDS) During Pregnancy Following In Vitro Fertilization (IVF)

**DOI:** 10.7759/cureus.57223

**Published:** 2024-03-29

**Authors:** Nika Vashakidze, Ana Mateshvili, Elene Pachkoria, Levan Ratiani

**Affiliations:** 1 Faculty of Medicine, Tbilisi State Medical University, Tbilisi, GEO; 2 Department of Infectious Diseases, The First University Clinic of Tbilisi State Medical University, Tbilisi, GEO; 3 Department of Anesthesiology and Reanimatology/Department of Infectious Diseases, The First University Clinic of Tbilisi State Medical University, Tbilisi, GEO

**Keywords:** medical managment, acute respiratory syndrome, pregnancy, in vitro fertilization (ivf), covid-19 infection

## Abstract

Coronavirus disease 2019 (COVID-19), the disease caused by the novel severe acute respiratory syndrome coronavirus 2 (SARS-CoV-2), is recognized for its heterogeneous clinical presentation, complex pathophysiology, and broad spectrum of manifestations. Obstetric patients have been a subject of considerable interest due to their potential vulnerability to more severe infection and adverse pregnancy outcomes, exemplified by this report of a 43-year-old pregnant female with severe COVID-19 infection and respiratory complications. This case report aims to contribute to the existing scientific knowledge by presenting a detailed clinical profile of a patient with COVID-19 who achieved pregnancy through in vitro fertilization (IVF) and discussing the management and outcomes of the infection. The neonate's negative COVID-19 tests suggest no intrauterine transmission; prompt medical interventions, including mechanical ventilation and targeted antibiotic therapy, were implemented to stabilize her condition. Close collaboration among the multidisciplinary team and careful monitoring of vital functions and laboratory parameters were crucial in managing the patient's complex condition. Thus, this case highlights the importance of a comprehensive and multidisciplinary approach in managing severe COVID-19 cases in pregnancy, emphasizing the need for prompt recognition of complications and timely intervention.

## Introduction

Coronavirus disease 2019 (COVID-19), the disease caused by the novel severe acute respiratory syndrome coronavirus 2 (SARS-CoV-2), is recognized for its heterogeneous clinical presentation, complex pathophysiology, and broad spectrum of manifestations. As of autumn 2021, there have been more than 250 million COVID-19 cases worldwide and more than five million deaths [1]. Obstetric patients have been a population of interest given that they may be at risk of more severe infection and adverse pregnancy outcomes. Upon initial presentation, approximately 95% of pregnant patients infected with COVID-19 showed no symptoms, and during follow-up, around 59% (with a 95% confidence interval of 49-68%) continued to remain asymptomatic [2]. The Delta variant (B 1.6.17.2) became the dominant strain of SARS-CoV-2 during the summer of 2021. There are several preliminary studies that have indicated increased disease transmission and severity associated with Delta infection during pregnancy [3]. Symptomatic pregnant individuals have a higher likelihood of experiencing rapid clinical deterioration. They are at an elevated risk of severe illness and death when compared to symptomatic nonpregnant women of reproductive age [4]. Physiological changes during pregnancy have a significant impact on the immune system, respiratory system, cardiovascular function, and coagulation. Risk factors that contribute to severe disease and mortality in pregnancy include advanced age (especially 35 years or older), obesity, preexisting medical conditions (especially hypertension, diabetes, or multiple comorbidities), and the absence of vaccination against SARS-CoV-2 [5].

The in vitro fertilization (IVF) technique is considered when natural conception proves challenging or unattainable. Miscarriage and obstetrical complications are potential risks that can impact pregnancies achieved through IVF, while neonatal prematurity and morbidity are more commonly associated with IVF neonates [6]. 

When it comes to COVID-19, viremia rates seem to be relatively low, as evidenced by a study showing around a 1% viremia rate [7]. However, it is worth noting that viremia might be higher in severe cases [8]. Despite these possibilities, the temporary and limited nature of viremia suggests that the transmission of the virus to the fetus and placental seeding are unlikely to be frequent occurrences during pregnancy [9]. The entry of SARS-CoV-2 into cells is believed to rely on the angiotensin-converting enzyme 2 (ACE 2) receptor and the serine protease TMPRSS2, and these two components are minimally expressed together in the placenta [10]. This limited coexpression in the placenta could explain why placental infection with SARS-CoV-2 and subsequent transmission to the fetus occurs infrequently.

This case report aims to contribute to the existing scientific knowledge by presenting a detailed clinical profile of a patient with COVID-19 who achieved pregnancy through IVF and discussing the management and outcomes of the infection. By analyzing individual cases, we can gain valuable insights into the unique challenges and considerations for managing COVID-19 in pregnant individuals.

## Case presentation

A 43-year-old pregnant woman, gravida 4, para 0, with a history of three previous spontaneous abortions, presented to the hospital via ambulance with a temperature of 37.8°C, productive cough, headache, runny nose, sore throat, and generalized weakness. The patient's current singleton pregnancy was achieved through IVF and she was at 22 weeks and six days of gestation. She had been receiving complete prenatal care, with no reported complications during the pregnancy. Past medical history was significant for hypothyroidism and left-sided scalping-oophorectomy done in 2015, due to a purulent process of unknown genesis. Her daily medications included folic acid, dydrogesterone (for miscarriage prevention), acetylsalicylic acid + magnesium hydroxide, and levothyroxine (100 mcg). Additionally, she had contact with a person who had COVID-19. The patient was diagnosed with COVID-19 three days after the onset of symptoms. Her COVID-19 vaccination status was negative. She initially received treatment at home under the care of her family physician, but her condition worsened requiring hospitalization.

Clinical course

Upon admission to the emergency department, the patient's vital signs were recorded as follows: temperature of 37.8°C, blood pressure of 115/70 mmHg, and oxygen saturation (SpO2) ranging from 91% to 94%. Physical examination revealed tachypnea with a respiratory rate of 22-24 breaths per minute. The laboratory results are given in Table 1.

**Table 1:** Laboratory Results

Investigation	Patient Value	Reference Range	Unit
C-reactive protein (CRP)	50	< 5.00	mg/L
WBC count (WBC)	6.04	4.00 - 11.00	10^9/L
Lymphocytes (LYMPH)	24.5	20.00 - 45.00	%
Granulocytes (GRA)	72.9	50.00 - 70.00	%
Platelet count (PLT)	214	150 – 380	10^3 µL
Hemoglobin (HGB)	11.5	12.5 - 15.5	g/dL
Troponin I	0.010	< 0.023	µg/L
D-Dimer	1.32	0.1 - 0.5	mg/L
Lactate dehydrogenase (LDH)	268	140 – 280	U/L
Interleukin-6 (IL-6)	64.1	<7.00	pg/mL
Ferritin	56.15	13.00 - 150.00	ng/mL

An obstetric ultrasound examination was performed, which revealed a fetal heart rate of 151 beats per minute with a regular rhythm. The placenta was posterior, with a thickness of 25 mm and a homogeneous structure. The placental maturity level was 0. The amniotic fluid was of normal amount and homogenous echotexture. The estimated fetal weight was 505 grams, corresponding to 22 weeks and five days of gestation.

The patient was admitted to the obstetrics-gynecology unit, The patient's condition continued to worsen despite initial treatment, as she developed a high fever (39.0°C) and increased shortness of breath. The patient’s hypoxia was resistant to oxygen mask therapy. Oxygen saturation dropped to 87% requiring a noninvasive continuous positive airway pressure (CPAP) and high-flow oxygenation initiation. 

On day 8, the patient was transferred to the intensive care unit (ICU) due to a worsening condition. The patient exhibited clinical signs of multi-organ dysfunction. Acute pneumonia developed in the context of the infection meeting the criteria for acute respiratory distress syndrome (ARDS). Despite the severity of the patient's condition, she remained conscious but periodically exhibited motor weakness. The patient's breathing was spontaneous, and gas exchange parameters were compensated with the support of oxygenation, non-invasive lung ventilation, and high-flow oxygen therapy.

**Figure 1:**
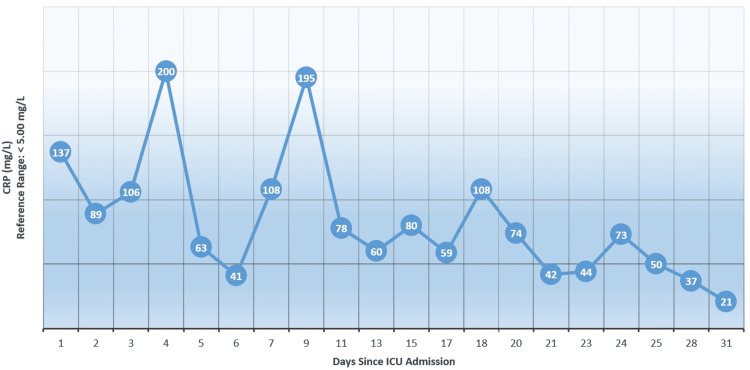
C-reactive protein (CRP) levels since ICU admission

**Figure 2:**
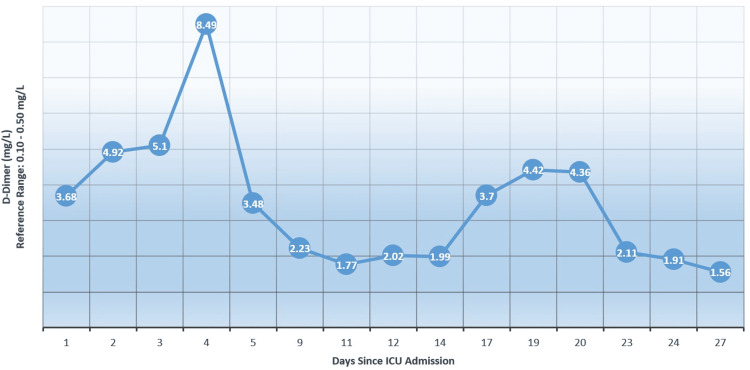
D-dimer levels since ICU admission

Laboratory tests conducted during the patient's stay in the ICU revealed worsening dynamics, with typical findings indicative of a cytokine storm. The IL-6 level increased to 633.6 pg/mL, C-reactive protein (CRP) rose to 106 mg/L (Figure 1), ferritin and lactate dehydrogenase (LDH) levels were elevated, and D-dimer levels remained increased (Figure 2). Due to further decompensation of the patient's respiratory function, non-invasive interventions were no longer sufficient to optimize gas exchange parameters. Orotracheal intubation was planned, and after proper medical preparation, the patient was transferred to mechanical ventilation on day 16 and tracheostomy was performed on day 27. Throughout the treatment, ultrasound monitoring of the fetus showed no evidence of fetal hypoxia.

The patient's condition remained severe, with critical respiratory failure, increased intra-abdominal pressure, and worsening laboratory indicators. Leukocyte and neutrophil counts increased, and CRP and ferritin levels continued to rise. Bacteriological research of sputum guided the administration of appropriate antibiotic therapy. The patient experienced high fever, and procalcitonin levels increased in correlation with other inflammatory markers. Radiological examination revealed fibrous-consolidation changes in the lungs. 

Due to the deepening respiratory failure and worsening clinical picture, a cesarean section was performed on the 25th day of hospitalization (24 weeks, three days). She delivered a 650-gram male infant with Apgar scores of 1/3/5; the newborn was transferred to the neonatal intensive care unit (NICU) for further treatment. The infant was not infected, COVID-19 test was negative. The patient's treatment continued in the ICU under the supervision of a gynecologist.

Treatment

Upon admission to ICU, the patient's treatment regimen was initiated with corticosteroid therapy, specifically intravenous dexamethasone at a dose of 8 mg per day. Subsequently, the patient's steroid therapy was transitioned to oral methylprednisolone at a dose of 16 mg per day. Antimicrobial therapy was initially commenced with meropenem and vancomycin, which was subsequently modified to moxifloxacin and colistimethate to ensure targeted treatment. To address potential gastrointestinal complications, gastroprotective therapy was administered intravenously using pantoprazole throughout the patient's hospitalization. Diuresis was managed by administering furosemide intravenously at a dose of 20 mg twice daily. The patient's thyroid function was supported by the administration of levothyroxine orally at a dose of 100 mcg once daily. In addition, the patient received supplementary therapy comprising B vitamins, vitamin C, antitussives, and albumin.

Outcome

Gradually, the patient showed signs of improvement, with neurological recovery, the establishment of communication, and the ability to follow simple instructions. Respiratory parameters improved, and breastfeeding was initiated. The patient was successfully removed from controlled breathing 37 days after hospitalization. Oxygen therapy was continued with a tracheostomy tube, and the patient became more active, participating in rehabilitative massages and exercises. The tracheostomy was later removed, and oxygen therapy was continued with a cannula at a flow rate of 3-4 L/min. The patient's respiratory parameters improved, with a respiratory rate of 16-17 breaths per minute and Spo2 levels of 98-99%. Hemodynamic parameters remained stable, with a blood pressure of 120/60 mmHg, heart rate of 80 bpm, and body temperature of 36.8°C. The patient's abdomen was soft on palpation, and vaginal discharge was not observed. The postoperative wound was appropriately managed and bandaged.

On the 42nd day, considering the patient's overall condition, she was transferred to the obstetrics-gynecology department, where treatment and monitoring continued according to the established protocol. The patient received infectious and anticoagulation therapies, with ongoing monitoring of vital functions. Water-saline and gas exchange corrections were performed based on arterial blood gas and electrolyte (ABG/EL) studies. Laboratory tests conducted during this period remained stable. Eventually, the patient was discharged from the clinic in a satisfactory condition under the supervision of her family doctor, with appropriate advice and prescriptions. The patient's follow-up care was coordinated with the pulmonologist, and she was referred for further management.

## Discussion

The impact of SARS-CoV-2 during pregnancy remains to be determined, and a concerted, global effort is required to determine the effects on implantation, fetal growth and development, labor, and neonatal health. Here, we reported the clinical course of COVID-19 infection in an IVF-achieved pregnant woman who was infected during the second trimester.

Dysregulation of iron homeostasis in COVID-19 contributes significantly to the pro-inflammatory state and the development of the disease. The elevated levels of ferritin, known as hyper-ferritinemia, observed in patients with SARS-CoV-2 infection are associated with iron toxicity. This is primarily due to the leakage of ferritin and the release of free iron from damaged tissues. Consequently, it is crucial to investigate iron metabolism in COVID-19 patients as it can serve as an important tool for monitoring the clinical progression of the disease and predicting a negative prognosis [11]. The elevated expression of ferritin in the placenta of mothers affected by COVID-19 may be attributed to the excessive activation of T lymphocytes and the increased activity of interferon‐gamma (IFN‐γ) during the inflammatory response. Previous studies have established a connection between ferritin and macrophage activation [12]. These iron-related processes play a significant role in the pathogenesis and severity of COVID-19. However, when considering obstetric patients, it has been observed that CRP serves as a more accurate inflammatory biomarker to assess the progression of the disease. Conversely, ferritin does not reliably predict an unfavorable outcome [13].

CRP levels are known to increase dramatically in response to injury, infection, and inflammation. CRP plays a crucial role in the acute-phase response that occurs after an inflammatory incident and is predominantly produced by the liver through interleukin (IL)-6-dependent synthesis [14]. Research indicates that CRP serves as a significant regulator of inflammatory processes, extending beyond its role as a mere indicator of inflammation or infection. CRP plays a vital role in various aspects of inflammation and the host's response to infection. These include involvement in the complement pathway, apoptosis, phagocytosis, release of nitric oxide (NO), and production of cytokines [15]. Increased levels of CRP could serve as a valuable early indicator for predicting disease progression in non-severe patients with COVID-19 [16]. In pregnant patients, the levels of CRP are associated with the need for medical intervention. The need for medical intervention in pregnant patients can be predicted with high accuracy using a CRP cutoff of 1.28 mg/dL on days 4-6 after the onset of symptoms [17]. Mothers who did not survive the infection displayed elevated levels of CRP compared to those who survived, suggesting a more severe infection and heightened inflammatory response. These increased CRP levels may also indicate the presence of an underlying cytokine storm in COVID-19. A cytokine storm is characterized by severe clinical manifestations such as acute respiratory distress syndrome, multiple organ dysfunction syndromes, and maternal mortality [18].

During pregnancy, D-dimer levels tend to rise, possibly due to the ongoing activity of the coagulation/fibrinolytic system during placenta development. Additionally, the increase in fibrin may be a consequence of blood stagnation in the lower limbs caused by the enlargement of the uterus [19]. Elevated D-dimer values at admission and during the peak of the condition in COVID-19 patients appear to be linked to deteriorating clinical outcomes, particularly an increased likelihood of requiring intubation and experiencing mortality. Patients who had an admission D-dimer of <2 ug/mL vs ≿2 mg/mL were observed to be intubated 15% vs 48.1% (P <.01) and mortality was noted to be 7.5% vs 40.7% (P <.01), respectively [20]. 

## Conclusions

We presented a case of a 43-year-old pregnant female who achieved pregnancy through IVF with severe COVID-19 infection and respiratory complications. Prompt medical interventions, including mechanical ventilation and targeted antibiotic therapy, were implemented to stabilize her condition; The neonate's negative COVID-19 tests suggest no intrauterine transmission; close collaboration among the multidisciplinary team and careful monitoring of vital functions and laboratory parameters were crucial in managing the patient's complex condition. Despite the challenges, including prolonged mechanical ventilation and critical anemia, the patient showed gradual improvement in her clinical status, including neurological recovery and successful weaning from controlled breathing. Thus, this case highlights the importance of a comprehensive and multidisciplinary approach in managing severe COVID-19 cases in pregnancy, emphasizing the need for prompt recognition of complications and timely intervention.
